# Insights into Digital MedicRehApp Maintenance Model for Pulmonary Telerehabilitation: Observational Study

**DOI:** 10.3390/healthcare12141372

**Published:** 2024-07-09

**Authors:** Michele Vitacca, Mara Paneroni, Manuela Saleri, Chiara Giuseppina Beccaluva

**Affiliations:** 1 Respiratory Rehabilitation Unit, Istituti Clinici Scientifici Maugeri IRCCS, 25065 Lumezzane, Italy; michele.vitacca@icsmaugeri.it (M.V.); mara.paneroni@icsmaugeri.it (M.P.); manuela.saleri@icsmaugeri.it (M.S.); 2 MedicAir Healthcare Telerehabilitation Service, 21140 Origgio, Italy

**Keywords:** chronic disease, respiratory tract diseases, telerehabilitation, digital health, home care services

## Abstract

Maintenance strategies after center-based pulmonary rehabilitation (CBPR) are currently needed. This study aimed to evaluate the feasibility and effect on the quality of life of a home-based pulmonary rehabilitation (HBPR) program delivered by a digital app. As secondary objectives, the patients’ adherence, symptoms, effort tolerance changes, and safety were evaluated. This was a single-arm prospective observational monocentric study on 30 patients referred for chronic respiratory diseases. The prescription and evaluations of the HBPR programs performed at the pulmonary facility and delivery of structured exercise and counselling by the digital health tool were achieved under the supervision of a respiratory therapist. Digital capabilities included aerobic, strength, and respiratory exercises, which were monitored with a fitness tracker. The engagement rate of the HBPR after the CBPR was 1:10. The EuroQoL VAS score increased from 66.2 ± 16.28 to 75.60 ± 16.07 (*p* < 0.001), mainly in younger subjects. No patient was lost during the HBPR program. The global adherence session rate was 94%. The Medical Research Council dyspnea scale (MRC), COPD Assessment Test (CAT) score, and six-minute walking test (6MWT) improved from admission into the pulmonary unit to the end of the HBPR program. Between the beginning and end of the CBPR, the CAT score decreased from 14.4 ± 6.39 to 8.50 ± 5.39 (*p* < 0.001), the MRC decreased from 1.87 ± 0.9 to 1.17 ± 0.83 (*p* < 0.001), and the 6MWT increased from 451 ± 93 to 473 ± 115 m (*p* < 0.05). The average Technology Acceptance Model score for usability was high (145 ± 12.1) and no adverse events occurred during the HBPR program. This HBPR model seemed to be feasible and well-accepted by patients, leading to improvements in quality of life, symptoms, and functional capacity.

## 1. Introduction

Center-based pulmonary rehabilitation (CBPR) is considered the gold standard intervention for functional recovery and quality of life improvement in patients affected by chronic obstructive pulmonary disease (COPD), asthma, interstitial lung disease, bronchiectasis, and post-COVID-19 condition [1]. The main benefits of CBPR programs are reached in terms of physical and psychological well-being, the person’s health, and quality of life [1]. However, benefits appear to decline after 6–12 months following the formal CBPR program [2], while the benefits in the health-related quality of life (HRQL) are better preserved than exercise performance [3] and are still present up to 2 years after the intervention [4].

Maintenance strategies to help with sustaining the benefits of CBPR are welcomed but contradictory results are available: whilst COPD patients that undergo repeated outpatient pulmonary rehabilitation (PR) programs do not show any significant worsening in exercise tolerance, dyspnea, and HRQL over a period of 7 years [5], other less-structured interventions after CBPR discharge (self-management, unsupervised home-based pulmonary rehabilitation [HBPR] programs, pathways conducted in community centers) generally fail to maintain these benefits [6,7,8,9,10], or produced only modest improvements [11]. In another randomized study, a home exercise program had no effect on dyspnea, but improved the 1 min sit-to-stand test performance and patient-perceived fitness, and the program was well accepted by patients with COPD [12].

As in other fields (e.g., the case of cardiac rehabilitation for patients with heart failure [13]), telemedicine could be adopted to support HBPR [14,15,16,17], and digital health tools (DHTs), such as wearables and mobile health devices, look promising for delivering physiotherapy and promoting healthier lifestyle, even in the long term [18,19]. Among digital solutions, smartphone and tablet applications could be the future of PR since a large proportion of respiratory patients now have access to the necessary technology and are using it properly [20]. Unfortunately, there is still sparse evidence about their efficacy and safety, and further research is needed to identify effective maintenance strategies with the adoption of real app-based medical devices instead of generic apps for fitness promotion.

This study aimed to evaluate the feasibility and effect on quality of life of a maintenance HBPR program after a CBPR course delivered by MedicRehApp^®^, which is a commercially available app for tablet and smartphone, under the surveillance of a hospital pulmonary facility and individually coached by a dedicated provider company (PC) respiratory therapist.

As secondary objectives, the study aimed to gauge participant responses, focusing on (a) patients’ adherence to the program, (b) medium-term symptoms and effort tolerance changes, (c) patients’ usability and satisfaction, and (d) safety.

## 2. Materials and Methods

### 2.1. Study Design and Population

This was a prospective observational single-arm study of patients admitted to the Pulmonary Rehabilitation Unit of ICS Maugeri—IRCCS Lumezzane (Lumezzane—BS, Italy) from September 2022 to December 2023.

The eligible subjects were patients discharged from a CBPR program; aged ≥ 18 years old; and with a primary diagnosis of COPD (Tiffenau index < 70%, FEV1 < 80%), asthma, bronchiectasis, pulmonary fibrosis, obesity (body mass index > 30), or post-COVID-19 condition. A mini-mental status examination > 22 and ability to perform a six-minute walking test (6MWT) with or without assistive devices represented additional requirements for inclusion.

The exclusion criteria were as follows: coexisting participation in other research projects or other rehabilitation interventions for any disease, high risk of heart failure and/or ventricular dysfunction, high thrombotic risk, planned surgery within 3 months after the study enrollment, high risk of arrhythmias, moderate-to-severe valve disease, instable respiratory disease, hemodynamic instability, anemia (Hb < 10 g/dL), pregnancy, drug abuse, total or partial inability to use digital devices, barriers to home exercise, and current all-day ventilotherapy.

### 2.2. CBPR Program

**Figure 1:**
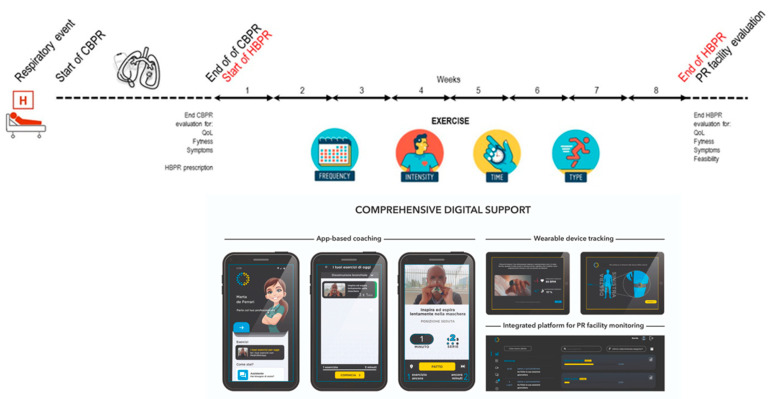
Patient journey according to the study protocol and home intervention. Abbreviations: CBPR—center-based pulmonary rehabilitation; HBPR—home-based pulmonary rehabilitation; PR—pulmonary rehabilitation; QoL—quality of life.

The CBPR program (Figure 1) was conducted in both residential and ambulatory settings. It was supervised by multidisciplinary teams of trained and experienced chest physicians, nurses, physical therapists, dietitians, and psychologists dedicated full-time to PR. It started within 2 days from admission, after baseline evaluations, and included daily supervised sessions (6 weekly days) of cycle training according to Maltais et al. [21] until performing 30 min of continuous exercise at 50–70% of maximal load, as calculated using the baseline six-minute walking test (6MWT) according to Zainuldin et al. [22]. The workload was increased by 5 Watts when the subjects scored their dyspnea or leg fatigue as <3 on a 10-point Borg scale, remained unchanged if the score was 4 or 5, and was reduced for scores of >5. Pulse oximetry, arterial blood pressure, and heart rate were monitored during sessions. The program also included strength training (upper limb, abdominal, and lower limb muscle activities by progressively lifting weights), and optimization of medications, education, nutritional programs, and psychosocial counseling when appropriate. The duration of daily activities was 2–3 h. The program was performed in a gym room with full availability of safety tools for cardiopulmonary resuscitation.

### 2.3. Maintenance HBPR Program

At the end of the CBPR program, all consecutive patients were offered a maintenance HBPR program managed by MedicRehApp^®^. The consecutive sampling technique was used to minimize the enrolling bias due to several clinical and logistic exclusion criteria. The intervention (Figure 1) was individually prescribed by the PC therapist in terms of exercise training (all patients), respiratory physiotherapy was given as needed (inspiratory muscle training, airways clearance techniques), and counselling was given as needed (for physical activity, respiratory techniques, oxygen therapy, aerosol therapy, and non-continuous ventilotherapy).

**Figure 2:**
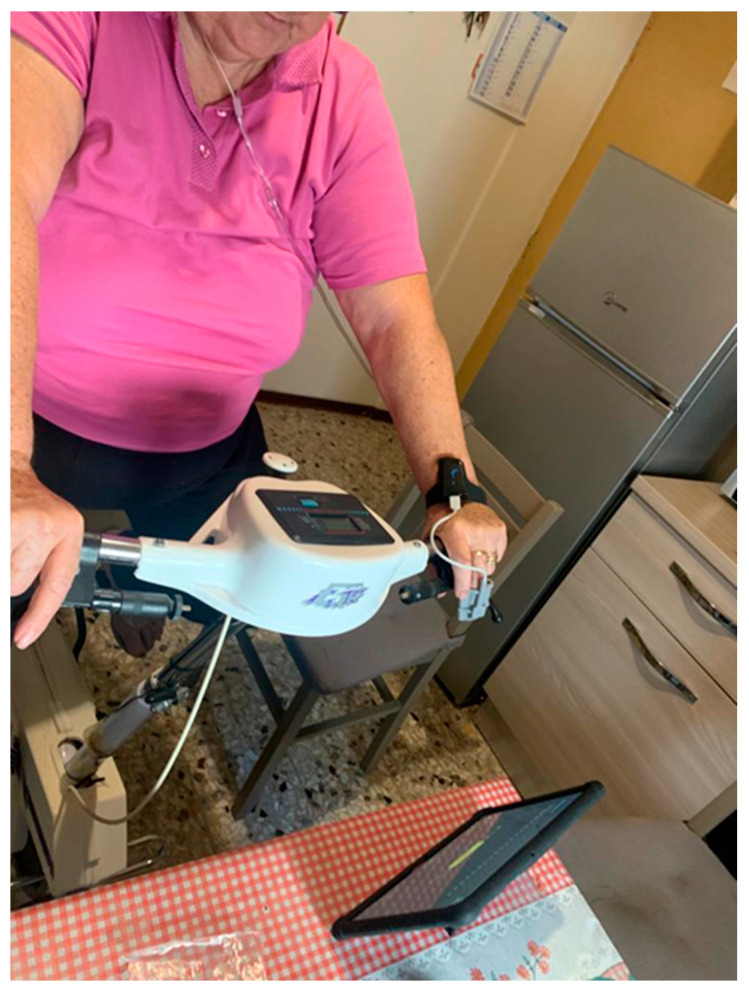
Representation of an at-home patient performing oxygen-supplied exercise guided by a tablet running the app and equipped with wearable devices.

Exercise training included both the aerobic and strength domains, with a structured prescription in terms of frequency, intensity, time, and type. Integrative training for flexibility, balance, and coordination represented other prescription options. Aerobic exercise was performed at home with a cycle ergometer or treadmill based on individual availabilities. Within 2 weeks after the prescription, patients were evaluated at home by the PC-trained respiratory therapist for logistic checks, DHT activation, device-focused counselling, definition of the exercise agenda, and in-presence supervised delivery of the first rehabilitative session. The following sessions (Figure 2) were then delivered both in remote synchronous (one per week) and self-managed asynchronous (four per week) modalities. The unique synchronous session per week was devoted to both aerobic and strength training, while out of the remaining four asynchronous sessions per week, two were for aerobic exercise and two for strength exercise. At the end of the prescribed 40-session program, patients were re-evaluated at the facility for the primary and secondary study end-points.

### 2.4. Digital Health Support

MedicRehApp^®^, which is a CE-certificated IIa class medical device (UDI-DI 805957590MEDICREHAPPAZ), is a comprehensive DHT for remote PR delivery that integrates mobile health technologies into home-based exercise training and lifestyle counseling. This DHT was developed by an industry partner (MedicAir Ltd., Origgio, Italy) who was not involved in the study design but participated in delivering parts of the DHT-based intervention, including direct patient coaching. Following individualized prescription at the facility, participants were provided with MedicRehApp^®^ and two wearable fitness trackers (CheckmeO2 max, Viatom Technology Co, Beijing, China; Movella DOT, Henderson, NV, USA) to self-manage their activity and share data with a dedicated respiratory therapist coach. The app was viewable on a dedicated tablet device, while the fitness tracker was composed of a pulse oximeter and an inertial motor sensor. Participants received a weekly telehealth-based visit from the respiratory physiotherapist, providing up- or downregulation of the exercise program in terms of structured education supported by multimedia contents. Patients were able to self-log exercise activity, vital signs, and medication adherence, as well as receive reminders for session execution via the app. An integrated online case management dashboard served for all patient and coach activities.

### 2.5. Primary Outcomes

The primary end-point for feasibility was the effective rate of subjects enrolled in the digital-based HBPR program out of all patients at the end of the CBPR and types of activity executed.

The primary end-point for efficacy regarding the quality of life was the variation between the beginning and end of the HBPR program, as measured by the EuroQoL VAS questionnaire [23].

### 2.6. Secondary Outcomes

The secondary end-points for adherence were assessed as the number of dropouts and the rate of sessions executed out of those prescribed during the HBPR.

The secondary end-points for symptoms and effort tolerance changes were assessed as variations between the beginning and end of the HBPR program, as measured by the COPD Assessment Test (CAT) questionnaire [24], the Medical Research Council (MRC) dyspnea score [25], and distances during the 6MWT [26].

The secondary end-points for usability and satisfaction were assessed by the Technology Acceptance Model (TAM) questionnaire [27], with a theoretical top score of 154 indicating the maximum degree to which a person believed that using a system would enhance their performance.

The secondary end-points for safety were the occurrence of adverse events during the training program.

### 2.7. Sample Size

For the primary end-point evaluation, considering a Minimal Clinical Important Difference (MCID) of 8 points at EuroQoL VAS according to previous validated experience [28], a standard deviation of 15 points, an alpha error of 5%, and a power of 80%, the estimated sample size [29] for the before–after study was 30 study participants in this single study group.

### 2.8. Statistical Analysis

Statistical analysis was performed using STATA 11 (StataCorp, LLC, College Station, TX, USA). The mean ± standard deviation was used to describe continuous variables, while the percentage was used to describe binary or categorical data. A chi-squared test was used to evaluate differences in non-continuous variables. A two-time pre-to-post-comparison was performed by the Wilcoxon test, while the repeated-measures comparison (T-1, i.e., admission to pulmonary unit; T0, i.e., beginning of the HBPR program; and T1, i.e., end of the HBPR program) was performed by the Friedman test.

To evaluate the association between the improvement in quality of life and the baseline conditions, we studied the risk of improvement by at least 8 points on the EuroQoL VAS questionnaire using an odds ratio evaluation. The baseline variables considered were sex, age less than 65 years, BMI less than 25, the presence of long-term oxygen therapy, the presence of bronchial obstruction (FEV1% < 80), and 6 min walking test < 470 m (median). A *p*-value < 0.05 was considered significant.

## 3. Results

Demographic and clinical data of 30 enrolled patients are summarized in Table 1. The majority of participants were males, middle-aged, overweight, and cases with COPD, while half of the cases were under long-term oxygen therapy (LTOT), with mild functional impairment that was both obstructive or restrictive, symptoms and disease impact, and mild quality of life impairment and effort tolerance.

### 3.1. Feasibility

**Figure 3:**
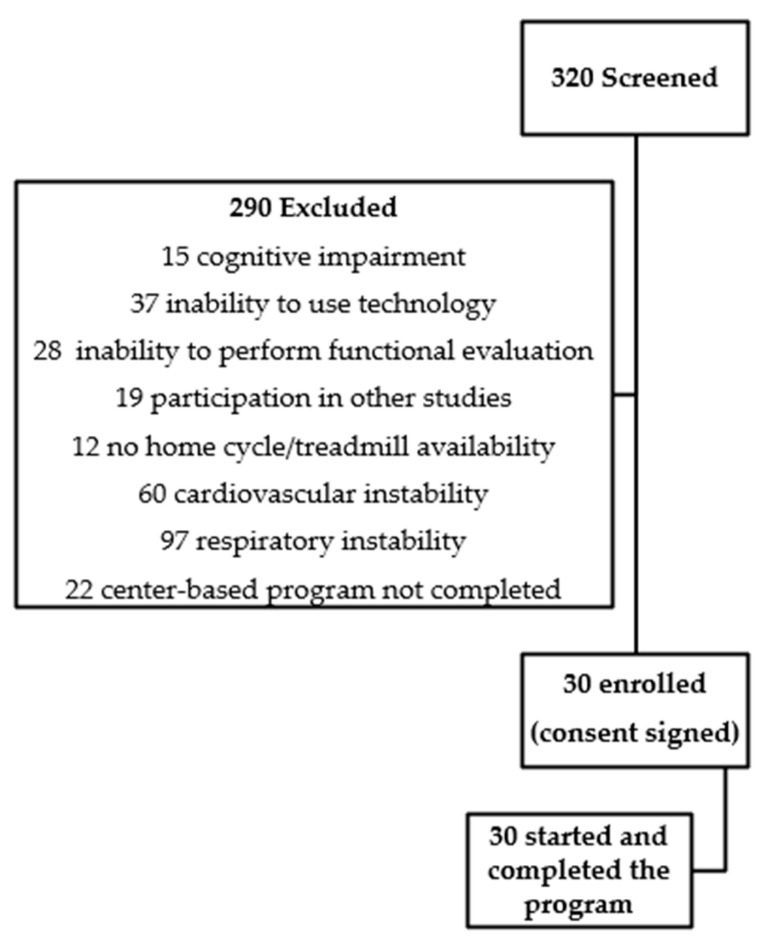
Summary of trial profile.

The rate of subjects enrolled in the digital-based HBPR program out of all patients at the end of the CBPR was about 1:10. Out of 320 individuals admitted to the PR facility with breathlessness and/or exercise limitation after an exacerbation, data from 290 individuals were excluded (Figure 3). The causes of exclusion were as follows: 15 with mini-mental status examination < 22, 28 were unable to perform a 6MWT, 19 were engaged in coexisting participation with other research projects, 28 had a high risk of heart failure and/or ventricular dysfunction, 12 had arrhythmias, 65 had unstable respiratory disease, 5 had hemodynamic instability, 15 had anemia (Hb < 10 g/dL), 37 had total or partial inability to use digital devices, 12 had barriers to home exercise, 3 had current all-day ventilotherapy, 29 had transferred to an acute care hospital or were deceased, and 22 had performed less than 12 sessions.

Concerning the performed activities, 40 sessions/pt and eight phone calls/pt were offered and all were executed. As a training modality, 22 patients used a cycle, 6 used a treadmill, and 2 walked. In 22 and 28 out of 30 patients, the loads for aerobic and strength training were increased to baseline, respectively.

### 3.2. Quality of Life

**Figure 4:**
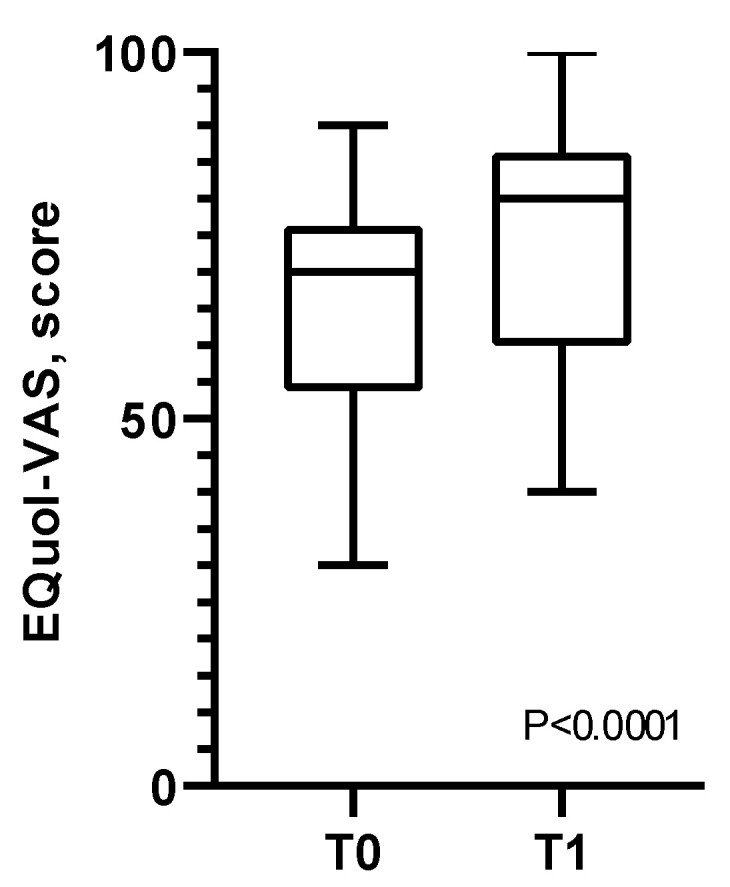
Variations of EuroQoL VAS score from the beginning (T0) to the end (T1) of the home-based pulmonary rehabilitation program.

At the final evaluation (T1), the EuroQoL VAS score increased from 66.2 ± 16.28 to 75.60 ± 16.07 (*p* < 0.001) (Figure 4). Younger people were more prone to improve their quality of life [OR 7 (1.36–35.92), *p* = 0.02] after the HBPR, while all the other baseline variables and conditions did not show any predictive power.

### 3.3. Adherence

No patient was lost during the HBPR program. The global adherence session rate was 94%, ranging from 55% to 100%.

### 3.4. Symptoms and Effort Tolerance

**Figure 5:**
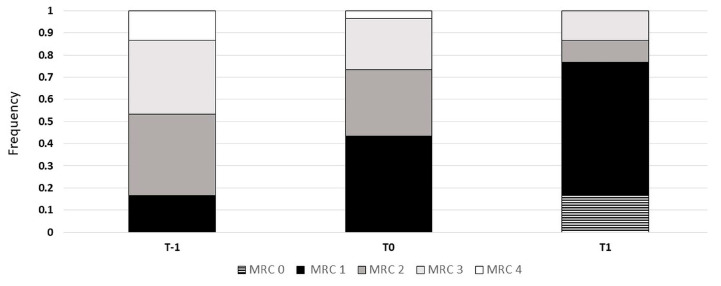
Variations in MRC score between beginning of center-based pulmonary rehabilitation (T-1), end of center-based pulmonary rehabilitation (T0), and home-based pulmonary rehabilitation (T1) programs (chi-squared *p* < 0.0001). Abbreviation: MRC—Medical Research Council.

**Figure 6:**
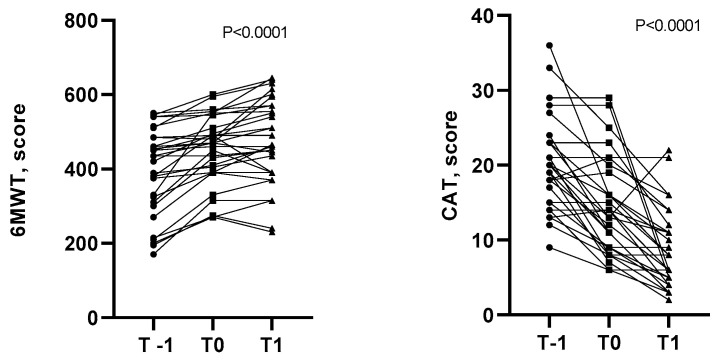
Variations in COPD Assessment Test score and 6 min walking test distances between beginning of center-based pulmonary rehabilitation (T-1), end of center-based pulmonary rehabilitation (T0), and home-based pulmonary rehabilitation (T1) programs.

The MRC score (Figure 5), CAT, and effort tolerance (Figure 6) statistically improved over time from the admission into the pulmonary unit (T-1) to the end of the HBPR program (T1). As shown in Figure 5 and Figure 6, at the end of the hospital program (24 ± 3 sessions), all patients improved their MRC score, while the majority improved their effort tolerance and CAT score. Between T0 and T1, improvements in symptoms and exercise tolerance were shown: the CAT score decreased from 14.4 ± 6.39 to 8.50 ± 5.39 (*p* < 0.001); the MRC score decreased from 1.87 ± 0.9 to 1.17 ± 0.83 (*p* < 0.001); and finally, the 6MWT increased from 451 ± 93 to 473 ± 115 m (*p*< 0.05), with an average gain of 22 m.

### 3.5. Usability and Satisfaction

At the end of the HBPR program, the average TAM score was 145 ± 12.1, with a minimum score of 105 and a maximum of 154.

### 3.6. Safety

No adverse events nor re-hospitalizations for acute exacerbations or other conditions were documented in the study group during the HBPR program.

## 4. Discussion

Our study showed that the HBPR program enhanced with a specifically designed DHT was feasible after a CBPR course, with high adherence rates and degrees of patients’ usability and satisfaction. Our combined CBPR + HBPR program improved the quality of life, symptoms, and exercise tolerance at the end of both the CBPR and HBPR.

Of note, the participants in our study were enrolled at the end of an intensive multicomponent residential PR cycle, and thus, were assumed as having little potential for further clinical improvement. These results referred to a hybrid program where exercise prescriptions and initial and final evaluations were made at the pulmonary facility, while the program implementation was entrusted to an external industry partner responsible for the patient’s engagement with the various DHT features.

If, on the one hand, this ensured a professional level of supervision on the use of the digital solution, on the other hand, a close collaboration was required between the referring facility and the program’s case manager, mainly for ongoing modulations of exercise and early detection of clinical instability. Moreover, other factors that favored our positive results were a well-educated and well-motivated patient population (having completed a previous CBPR cycle), and the absence of out-of-pocket expenditures with comprehensive DHT supply and service. Notwithstanding these favorable situations, the engagement rate of the HBPR program after the CBPR was limited to 1:10 due to several individual, clinical, and logistical conditions. These features need to be considered when translating this operation modality to different settings and situations.

Evidence on digitally enhanced PR is scant and mixed for the solutions evaluated. In 2017, Bourne et al. [30] found that a 6-week program of online-supported PR (“myPR”, incremental in nature and led by a physiotherapist) was non-inferior to a conventional out-patient model with face-to-face sessions in terms of functional capacity and symptoms, and at the same time was safe and well tolerated.

Concerning mobile applications for the delivery of exercise training, they generally reflect an early stage of development, and above all, different capabilities in terms of incorporated automatic recording and data syncing during exercise, real time feedback, and correctional goal setting. For PR purposes, the initial findings were disappointing. In 2016, Vorrink et al. [31] performed a randomized controlled trial in 32 physiotherapy practices in the Netherlands on 121 COPD patients with the adoption of a smartphone application and a monitoring website for the physiotherapists without significant positive effects on physical activity at 12 months. Recently, Spielmanns et al. [32] tested the Kaia COPD app in the post-rehabilitation phase for the maintenance of physical activity in patients with COPD, with positive results in terms of daily steps and symptoms. This German-speaking tool was able to progressively increase the exercise activity based on patient feedback recorded through the app and the number of steps per day was collected by an activity tracker. Our study was conducted in a similar post-residential PR setting and with the adoption of both an app and a fitness tracker, and thus, with similar “digital power”, and seemed to confirm these results.

The direct comparison of the app-based HBPR and CBPR as different alternatives underwent a systematic review published in 2024 [33], including nine studies and with various formats of mobile apps used. Five DHT interventions included exercise adjustment, seven included contact with a healthcare professional, and six included disease monitoring. Newly developed apps were adopted in some studies, while others used myCOPD (also providing education and symptom management programs) or the social messenger app WeChat, providing only exercise programs. Globally, app-based PR showed favorable exercise capacity, symptom score, quality of life, and hospitalization rates when compared with traditional CBPR. Distances for the 6MWT, MRC scores, and exacerbations in the app-based PR group were not inferior to those in the CBPR group, and the CAT scores were even superior. However, these data were obtained by studies with small sample sizes and did not provide information on app usage, and therefore, more information is needed.

### 4.1. Strengths and Limitations

The strengths of our study were the focus on feasibility and quality of life—which is a strong indicator of quality of care in respiratory patients—and the adoption of direct testing (i.e., the 6MWT) for the evaluation of exercise tolerance in spite of the daily steps count or questionnaires exploring daily physical activity habits. Furthermore, an operational flowchart that includes active patient’s coaching by health professionals gives the possibility to integrate exercise supervision with behavioral and psychosocial support (e.g., adherence to pharmacotherapy, ventilation, and lifestyle; anxiety and depression management; returning to work) closer to the delivery of main core components of comprehensive PR. 

However, several limitations need to be considered. First, the patient population was variegated and small, even though it was appropriate according to preliminary sample size evaluation and in line with other app-based pilot projects. Second, the absence of a control group represents a barrier for result assessment. However, the positive results we found indicated a net clinical benefit and probably countered the natural regression of PR benefits in the absence of maintenance [2]. Third, our results may not be generalizable to the whole population of respiratory patients after PR irrespective of center-based metrics (particularly for enrollment rates to HBPR), baseline levels of functional capacity, results of the PR program, patient-related barriers, and effective adherence to the DHT. Finally, the direct and indirect costs were not fully evaluated.

### 4.2. Practical Implications

The adoption of rehabilitative tablet and smartphone applications offers a “digital therapy” solution that contributes to integrate regular exercise training into patients’ daily life, increasing the access opportunity and facilitating PR maintenance strategies. The promising preliminary results indicate that the proposed intervention is poised for examination in a more extensive trial. This model, which was favorably received by patients, holds the potential for mitigating the hospital burden and barriers to rehabilitation, especially for individuals living in challenging geographical areas. It is vital to thoroughly explore the viewpoints of various stakeholders and ensure appropriate governance of such joint ventures between public and private institutions as far as reimbursement policies are concerned [34].

## 5. Conclusions

Based on this study, the combined CBPR and HBPR approach enhanced with a specifically designed DHT demonstrated feasibility, reliability, and acceptability, leading to a notable improvement in quality of life, in particular for younger subjects. Randomized, controlled studies on large patient populations are warranted to further assess the quality standards, cost-effectiveness, and long-term efficiency of such an approach.

## Figures and Tables

**Table 1:** Demographic and clinical data of the study population.

Measures	
Patients, n	30
Male, n (%)	20 (67)
Age, years	61.93 ± 10.62
BMI, kg/m^2^	27.67 ± 7.16
Diagnosis	
*COPD, n (%)*	*16 (53)*
*Asthma, n (%)*	*4 (13)*
*Bronchiectasis (%)*	*5 (17)*
*Obesity, n (%)*	*4 (13)*
*Post-COVID-19, n (%)*	*1 (3)*
LTOT, n (%)	14 (47)
FEV1, % predicted	67.13 ± 29.19
FVC, % predicted	86.89 ± 23.27
FEV1/FVC	74.77 ± 23.21
MRC, score	1.87 ± 0.90
CAT, score	14.4 ± 6.39
EuroQoL, score	6.20 ± 2.02
EuroQoL VAS, score	66.17 ± 16.28
6MWT, meters	451.33 ± 93.10

Data are expressed as number, percentage, or mean and standard deviation. Abbreviations: 6MWT—6 min walking test; BMI—body mass index; CAT—COPD Assessment Test; COPD—chronic obstructive pulmonary disease; FEV1—forced expiratory volume in the 1st second; FVC—forced vital capacity; LTOT—long-term oxygen therapy; MRC—Medical Research Council; VAS—visual analytic scale.

## Data Availability

Original data are available from the corresponding author upon reasonable request.
